# Future of Hyatt Regency Maui’s African Penguin Colony: An Analysis of Stakeholders

**DOI:** 10.1177/21649987241290983

**Published:** 2024-10-18

**Authors:** Danielle Duffhues, Prescott C. Ensign

**Affiliations:** 1 Wilfrid Laurier University, Waterloo, ON, Canada

**Keywords:** hospitality and tourism, stakeholder theory and analysis, stakeholder salience model, endangered species

## Abstract

This case provides students with an opportunity to use a stakeholder theory framework to analyze the interests and influence that various stakeholders have on Hyatt’s African penguin colony. Hyatt’s penguin colony has delighted guests and visitors for over 40 years. In order for the colony to survive, four African penguins need to be imported for breeding. Unfortunately, the Hawai‘i Department of Agriculture’s Executive Board did not grant Hyatt’s request for an import permit. Evidently, changing attitudes by government officials and certain interest groups toward tourism and related issues impacted their decision. What should Hyatt management do?

## Case Study Learning Objectives

By the end of this case study, students should be able to:

1. Explain the stakeholder theory framework as it relates to management decision-making.
2. Identify the challenges, ethical issues, and responsibilities management faces from internal and external stakeholders regarding its decision on the future of its African penguin colony.
3. Apply the stakeholder salience model (see Figure 2) to analyze and critique stakeholders regarding Hyatt’s request to import four African penguins for its colony.
4. Formulate and defend a stakeholder-based strategy that Hyatt could use to resolve the dilemma for the endangered African penguin colony.

## Introduction

The Hyatt Regency Maui Resort and Spa (Hyatt) has had a small colony of African penguins on display for the past 40 years. The future of this colony was dependent on adding at least four penguins for breeding to support the colony. Hyatt arranged with International Animal Exchange, Inc. of Royal Oak, Michigan to obtain these penguins. In order to obtain these penguins, an import permit needed to be obtained from the Hawai‘i Department of Agriculture (HDOA) (Carisa-Abney, 2022, p. 2). Hyatt prepared technical documentation to support its request in November 2021. HDOA’s Advisory Committee approved Hyatt’s request in May 2022. Final review and approval had to be made by the ten-member HDOA Executive Board. At a meeting on August 23, 2022, the Executive Board did not approve Hyatt’s request for an import permit. Changing attitudes by government officials and certain interest groups toward tourism and related issues impacted the decision on the penguin colony. This was surprising since tourism was Hawai‘i’s largest industry and the impact even larger on Maui where 51% of jobs were associated with serving visitors (Maui County, 2017). A stakeholder theory framework is introduced to analyze Hyatt’s stakeholder groups. Using this approach helps management formulate a strategy to resolve issues like the dilemma facing Hyatt’s penguin colony.

## Hyatt’s African Penguin Colony

The Hyatt Regency Maui Resort and Spa, a luxury hotel established in 1980 with over 800 guest rooms, was located in Lahaina on 40 acres of land on Kā‘anapali Beach on the island of Maui. The popular tourist destination was AAA Four Diamond-rated (Hyatt Regency Maui, n.d.). Hyatt’s small colony of African penguins began in 1983. The only other place in Hawai‘i that African penguins could be seen was at the zoo in Honolulu on the island of O‘ahu (Carisa-Abney, 2022: Attachment 4, p. 5). There were 18 species of penguins still in existence, 5 native to warmer non-Antarctic coastal regions. None were endemic to Hawai‘i. African penguins could reach up to 60–68 cm (24–27 inches) in length and weigh up to 3.7–4 kg (8–9 pounds) with males being slightly larger than the females (Britannica, 2024). Like many other bird species, African penguins were monogamous and pairs mated for life (SANBI, 2018). In 2010 African penguins were placed on both the international (IUCN, 2020) and US federal list of endangered species (US Fish and Wildlife Service, 2010). The Association of Zoos and Aquariums (AZA)/Penguin Taxon Advisory Group (TAG) recommended a minimum of 10 penguins in an exhibit to support the necessary genetic and social structure (AZA Penguin Taxon Advisory Group, 2014). Clearly, the future existence of this colony of six African penguins was at a genetic and breeding tipping point.

The current six penguins (three females and three males) were in a habitat just off the atrium lobby. All birds were bred at the Hyatt—twins Mai and Tai as well as Nahu, Buddah, Zen, and Momi (the youngest at age 10). There had been no births since Momi (Kā‘anapali Beach Resort, 2024). The design and operation of the penguin habitat was in compliance with the newly enacted US Animal Welfare Act license and registration standards for birds (APHIS, 2024). Hyatt followed AZA and TAG standards to protect the specific sustainability and breeding needs of African penguins. The habitat was large enough to accommodate 20 birds and staffed by a qualified wildlife supervisor, technician, and an on-call veterinarian (Carisa-Abney, 2022, p. 3). The staff also took care of other wild bird species at the Hyatt including cranes, flamingos, swans, and ducks. Each penguin consumed about 100 pounds of small fish every week. Hotel guests, especially children, enjoyed watching them eat, swim, sunbathe, dig burrows in the sand, and waddle around. Hyatt also offered guests and visitors a free educational program about African penguins, including their plight as an endangered species. Every morning at 9:30 am this free presentation was conducted in the penguin habitat adjacent to the atrium lobby (Kā‘anapali Beach Resort, n.d.).

## Future of the African Penguin Colony

Hyatt submitted a request to HDOA to import four African penguins on November 5, 2021. Since penguins were on the state’s list of restricted animals, they could only be imported by permit and for exhibition. Hyatt had planned to import the penguins through the International Animal Exchange, Inc. (Royal Oak, Michigan). As Hyatt’s application stated: “Currently, our facility can no longer produce viable offspring as they are too closely related. We are seeking to obtain 4 penguins of a different bloodline to ensure genetic vitality” (Carisa-Abney, 2022, p. 2). Hyatt’s request was thoroughly reviewed by the Advisory Committee of the HDOA’s plants and animals quarantine branch. This Advisory Committee approved Hyatt’s request in May 2022, provided pre-entry health screening, and initial quarantine protocol was followed (Carisa-Abney, 2022).

The Executive Board of HDOA made final decisions on import permits. The ten-member board consisted of: one representative from each of the counties (Hawai‘i, Kaua‘i, and Maui); three at-large members; and four members based on their professional position (Chairperson of the Department of Agriculture, Chairperson of the Department of Land and Natural Resources, Director of the Department of Business, Economic Development and Tourism, and Dean of the University of Hawai‘i’s College of Tropical Agriculture and Human Resource; HDOA, 2024).

The HDOA Executive Board voted on Hyatt’s request on August 23, 2022. This was a virtual meeting (mixed phone and video) and the only person speaking in favor of importation was Hyatt’s Wildlife Supervisor, Povi Carisa-Abney. She had prepared Hyatt’s request (394-page document) and presented it in person to the Advisory Committee. Since the Executive Board meeting also had a full agenda of other items it was several hours before they considered Hyatt’s request (YouTube, 2022). The 10 Board members voted: 5 for, 4 against, and 1 abstention. Hyatt’s request did not get the necessary six votes to pass or six votes to be rejected thus tabling the request (HDOA, 2022). Hyatt acted as though getting an import permit would not be a problem since they had received approval to import five African penguins in 1992 (HNN, 2021). Also, the Honolulu Zoo had recently received approval (2021) to import four female African penguins from zoos on the mainland (Van Dyke, 2021) and Maui’s Grand Hyatt Kauai Resort had received approval to import two exotic mute swans native of Eurasia (Honore, 2022). What went wrong? A stakeholder analysis is introduced to help Hyatt personnel understand the opinions and influence for this vote and formulate a response.

## Stakeholder Analysis Framework

**Figure 1.**
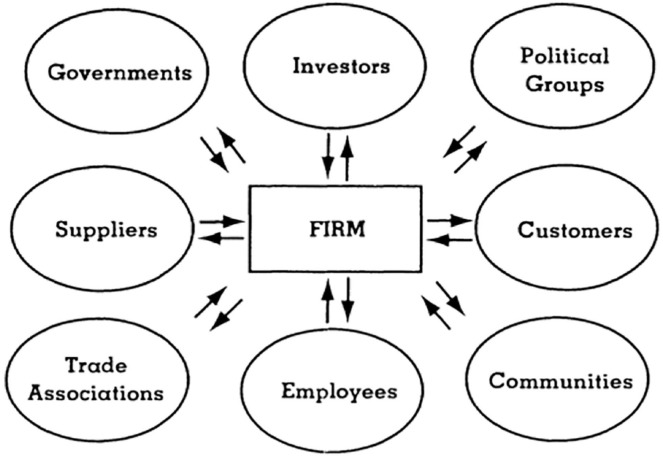
The stakeholder theory model. *Source.* Donaldson and Preston (1995, p. 69).

Hotels have a complex array of competing interests, relationships and influences that must be effectively managed (Font & Lynes, 2018). Stakeholder theory presents a framework that hospitality and tourism managers can use to classify and prioritize internal and external groups on issues such as the future of Hyatt’s colony of African penguins. A stakeholder approach identifies and models *which* stakeholders really count as well as *why* and *how*. Traditionally, management viewed stakeholders as owners or shareholders. Stakeholder theory argues that there are many groups that impact and are impacted by the business (Freeman, 1984). Figure 1 provides an example of some stakeholder groups that can impact managerial actions on a given issue.

### Review of Literature

Barakat and Wada (2021) reviewed the use of stakeholder theory in the hospitality industry literature published from 1984 to 2018. Some of the important benefits were: insight into stakeholder interests and influences; consideration of economic, social, and ethical issues; a focus on relational rather than transactional linkages; and provides a strategic framework for decision-making. As Barakat and Wada (2021), Nicolaides (2015), and Font and Lynes (2018) note, there have been few empirical studies of stakeholder theory in the hospitality and tourism field. There are some studies that focus on: stakeholder engagement (Coles et al., 2013); corporate social responsibility (Farmaki & Farmakis, 2018; Font & Lynes, 2018), sustainability (Sharpley, 2009), destination marketing organizations (Cooper et al., 2009; Sheehan & Ritchie, 2005), and customer engagement relative to firm performance (Elgarhy, 2023).

Most of these studies build on the work of Donaldson and Preston (see Figure 1) who suggest that stakeholder theory has three distinct approaches. Although these three are different, they are mutually supportive (Donaldson & Preston, 1995, pp. 66–67).

***• Normative Approach*** focuses on “*What should happen?*” regarding an organization’s stakeholders. The organization is viewed as an open system with a democratic and pluralistic platform where stakeholders are dealt with in a moral and ethical manner (Šmaguc, 2022, p. 87).
***• Descriptive Approach*** seeks to clarify “*What actually happened?*” in a given situation. It examines an organization’s characteristics, strategies, and positive and negative actions that influence and/or are influenced by different stakeholders (Jawahar & McLaughlin, 2001). This approach provides a useful framework for predicting an organization’s future actions (Friedman & Miles, 2006).
***• Instrumental Approach*** focuses on identifying stakeholder influences relative to operational choices, that is, “*What will happen if …?*” (Šmaguc, 2022). It is concerned with examining the relationship between the pressures of different influential groups, the way an organization formulates its strategy and impact on performance.

### Stakeholder Analysis and Mapping

Theoretically, all stakeholders matter. However, as Farmaki and Farmakis (2018, p. 58) found in their study on the implementation of corporate social responsibility (CSR) by hotels in Cyprus, there is a need to delineate among stakeholders. The need for management to classify and prioritize stakeholders requires taking an instrumental approach. The stakeholder salience model (Mitchell et al., 1997) can be used by management to evaluate a stakeholder’s comparative significance based on three discernable attributes.

**• Power**—The ability to influence groups or the organization to act in a certain way it would not have otherwise. The bases of this power can be coercive (force or threat), utilitarian (material or incentive), or normative (symbolic influence).
**• Legitimacy**—The extent to which a stakeholder has contractual, legal, or ownership claims. The perception that their actions are desirable, proper, or appropriate within some socio-politically constructed system of norms, values, or laws.
**• Urgency**—The degree to which a stakeholder can get immediate attention by the organization. This is either time sensitivity (the degree to which managerial delay in responding is unacceptable to a stakeholder) or criticality (the importance of the content or relationship to the organization).

**Figure 2.**
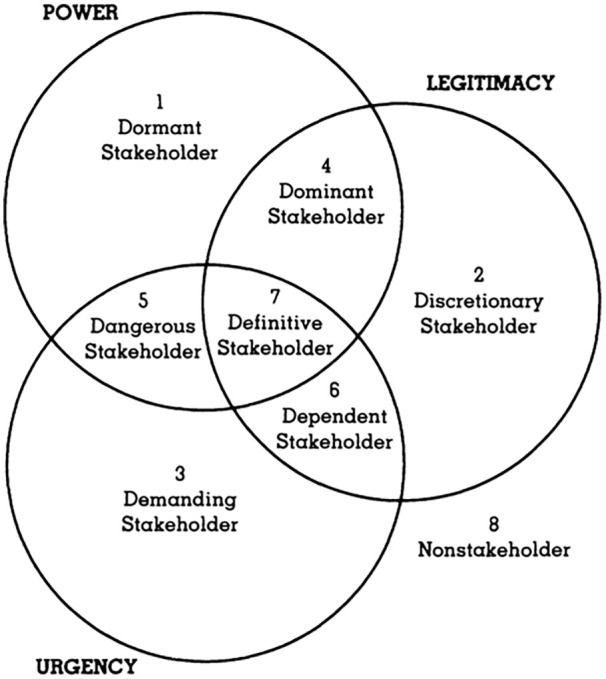
Stakeholder Analysis Using Salience Model. *Source.* Mitchell et al. (1997, p. 874).

The salience model provides a way to rank stakeholders based on the three attributes. Stakeholders that are less important have only one of the attributes. Those that are the most important possess two or three of the attributes. The salience model is an application of the instrumental approach (*what will happen if …*) to stakeholder theory. It contributes to effective stakeholder management by explaining how managers should prioritize their stakeholder relationships. A stakeholder analysis does not preclude overriding the interests of one group of stakeholders by the interests of another. It does, however, ensure that all those affected will be considered by management (Okumus et al., 2020). Figure 2 illustrates how managers can use stakeholder theory to map or categorize the salience or importance of a stakeholder based on three attributes (Mitchell et al., 1997, p. 869):

As illustrated in Figure 2, using the three attributes to categorize stakeholders results in eight types of stakeholders (Stareva, 2019).

**1. Dormant stakeholders**: Those with power but not urgency or legitimacy to exercise influence. Management should be aware of and monitor them but there is no need for major communication and involvement.
**2. Discretionary stakeholders**: They are legitimate but have no power or urgency. Their communication needs may be in the form of actually asking for some details but may not need a lot of attention other than just regular updates.
**3. Demanding stakeholders**: Those that always seem to think that they need immediate attention. If too much time and energy is spent on these stakeholders, the effort will not gain much traction. There are likely other more important stakeholders to work with, but these demanding stakeholders should be kept informed.
**4. Dominant stakeholders**: Power and legitimacy overlap in this instance. Dominant stakeholders have legitimacy and authority. Their communication and involvement needs must be considered at all times.
**5. Dangerous stakeholders**: These stakeholders have the volatile mix of power and urgency. This combination makes them crucial for the success of the effort as these dangerous stakeholders are in a position to have a negative impact. Therefore, management must meet their needs and keep them engaged and satisfied.
**6. Dependent stakeholders**: They are legitimate and have the urgency but do not have commensurate power. Keep them informed as they could be of help when the effort needs to leverage their strengths in dealing with problems.
**7. Definitive stakeholders**: The most important area in this model—where power, urgency, and legitimacy converge. This is the most critical category of stakeholders. Management must always keep these stakeholders informed, satisfied, and involved. Management needs to provide focused attention to these stakeholders.
**8. Non-stakeholder**: Regarding this issue, activity, or effort these non-stakeholders are not affected and do not affect the organization.

In summary, the highest priority stakeholders are those in the groups with all the attributes—number 7. Next in priority are those with any mix of 2 attributes—numbers 4, 5, and 6. The lowest priority stakeholders are those that have only 1 attribute associated with them—numbers 1, 2, and 3. Although management may decide that they have little importance, they need to be recognized because they could move to other groups. This is consistent with the findings of Farmaki and Farmakis (2018). Management needs to consider an inclusive approach so that community and special interest groups are not marginalized to the detriment of the overall sustainability of the tourism industry.

## Stakeholders That Impact Hyatt’s Penguin Colony

### Hawai‘i Department of Agriculture Executive Board and Its Members

Dr. Ania Wieczorek, Dean of the University of Hawai‘i‘s College of Tropical Agriculture and Human Resources abstained because she was a new board member and did not receive the application materials (YouTube, 2022). She did, however, publicly express reservations against importing exotic animals into Hawai‘i for commercial use and entertainment. Her position was similar to others who voted against Hyatt’s request (Smith, 2022).

One of the four who voted against Hyatt’s request was Diane Ley. She was the representative for the Island of Hawai‘i and served as Executive Director for the US Department of Agriculture’s Farm Service Agency in Hawai‘i and the Pacific Basin. In the Board meeting, Ley stated:

It sounds like the Hyatt has done a good job over the years taking care of the penguins. Nonetheless, the request is for entertainment – and any educational value to keeping them there is a stretch. Further, the Hyatt request to sustain the exhibit really raises the conflict of the trend that’s going on here in Hawai‘i with the public. Our public policy is shifting, and it’s shifting rapidly to encourage authentic local and Hawai‘ian practices and experiences to better inform visitors of what it means to be in this place (Honore, 2022).

James Gomes, member-at-large, questioned what tourists take home from their visit. His comment was: “What did I learn when I was here in Hawai‘i nei, in Maui? I looked at penguins” (Smith, 2022).

Another Board member voting against Hyatt’s request was Suzanne Case. Her position on the Board was based on her professional position as chair of Hawai‘i’s Department of Land and Natural Resources. In a later interview with *Civil Beat*, Case stated that everyone voted to deny the Hyatt request because “there is interest on the Hawai‘i side in portraying Hawai‘i in a genuine fashion. Exotic animals are not that. Many people are obviously interested in seeing exotic animals around the world, but are we doing that as an educational experience or as an entertainment experience?” (Honore, 2022).

One of the five who voted to support the request was Randy Cabral. He was a Hawai‘i island native and president of the Hawai‘i Farm Bureau Federation. Cabral noted the Board’s inconsistency in granting import permits. He stated: “Just last year the Board approved a request by the Grand Hyatt Kaua‘i Resort to import two mute swans. I don’t see the difference between these two requests. Why one and not the other?” (Honore, 2022).

### Supporting Interest Groups

Both the Hyatt Regency Maui Resort and Spa and the Hyatt Hotels Corporation supported the continued viability of the African penguin colony. Management committed funds and staff time to the penguin colony. Hyatt’s Wildlife Supervisor, Povi Carisa-Abney, was dedicated to the future of the penguin colony. She had responsibility for carrying the project forward. Globally, groups and individuals provided support for endangered species such as these iconic black-footed penguins. There were also public and private groups that recognized that any breeding efforts, even for animals in captivity, were beneficial for the continuation of the species. Hyatt guests and other visitors to the penguin colony continually dispensed positive feedback. Many hotels and businesses throughout Hawai‘i, as well as Hawaiian Airlines, encouraged tourist to visit the free African penguin exhibit. There was no doubt that Hyatt had the support of the 700+ member Hawai‘i Lodging and Tourism Association that served as a powerful advocacy and lobbying group.

### Opposing Interest Groups

Over the years, there had been many to question the legitimacy of public exhibitions of animals based on issues of exploitation, abuse, and poor care (Stainton, 2023). Some of the same concerns were raised about Hyatt’s penguin colony, abusing and exploiting the penguins for entertainment and financial gain. Others raised concerns that an imported animal could become invasive and detrimental to indigenous species if it escaped. This seemed unlikely since Hyatt’s biosecurity measures had never been thwarted. Also, African penguins could not survive in the wild without a supply of cold ocean water small fish (Carisa-Abney, 2022, pp. 8–9). Perhaps the greatest concern was that the COVID-19 pandemic increased public fear of disease spreading from animals to humans (zoonosis).

COVID-19 also had a huge impact on attitudes in Hawaiʻi toward tourism. Local residents discovered that they enjoyed life without tourists during the pandemic. Many were unsure if they wanted tourists to return (Terrell, 2020). This was reflected in the concerns of three groups. The first were Animal Rights Hawaiʻi. As president of this organization, Cathy Goeggel testified at the HDOA Executive Board meeting, that her group opposed Hyatt bringing in birds native to southern Africa. Goeggel said: “They do not belong in Hawaiʻi they do not represent Hawaiʻi. Animals are not ornaments … it is improper for this to continue. Just think about the image that presents to people who are visiting us. We have plenty of wonderful things for our tourists to see.” (HDOA, 2022). A second and more powerful group funded by the state’s Hawai‘i Tourism Authority was the Mālama Hawai‘i. A political and socio-cultural campaign to reverse decades of recreation-focused branding. Its goal was to attract “more thoughtful tourists” who respected the traditions of Hawai‘i. This promotional effort had not gained traction with tourists (Teruya & Yerton, 2022). A third was a pro-indigenous group, ‘Āina Momona. It was a non-profit grassroots community-focused and land resource conservation organization dedicated to achieving environmental health and sustainability for native flora and fauna through restoring social justice and Hawaiʻian sovereignty by decolonization (‘Āina Momona, n.d.).

## Challenges and Dilemma—Where Things Stand

As of late-2024, the Hyatt African penguin colony still needed additional birds if it was to remain viable. The HDOA Executive Board’s inconclusive vote on August 23, 2022 essentially tabled indefinitely Hyatt’s request to import four African penguins. Since the Board had not placed the item on an agenda for over a year, it seemed unlikely that it would. It remained for Hyatt Regency Maui Resort and Spa management and Hyatt Hotels Corporation executives to decide. Should they fight to save the penguin colony, wait for the penguins to age out, or shut down the exhibit and transfer the penguins? What should they do? What would you do?

## Discussion Questions

**1.** What is the stakeholder theory of organizational management?
**2.** What challenges, ethical issues, and responsibilities with stakeholders does the African penguin colony create for Hyatt management?
**3.** Assume that Hyatt management has assigned you the task of using the stakeholder salience model (see Figure 2) to analyze, critique, and map interest groups regarding the import of African penguins. What is your analysis? Explain.
**4.** Based on stakeholder theory, what would you recommend that Hyatt management do to resolve the African penguin colony dilemma? Defend your proposal.

If you are an ICHRIE member, you can access the Teaching Notes for this case study here: https://ichrie.memberclicks.net/jhtc. If you are not an ICHRIE member, the Teaching Notes will be published in a future Sage Business Cases (SBC) annual collection: https://sk.sagepub.com/cases. For more information, please contact info@sagepub.com
